# Soft-mode driven polarity reversal in ferroelectrics mapped by ultrafast x-ray diffraction

**DOI:** 10.1063/1.5026494

**Published:** 2018-04-06

**Authors:** Christoph Hauf, Antonio-Andres Hernandez Salvador, Marcel Holtz, Michael Woerner, Thomas Elsaesser

**Affiliations:** Max-Born-Institut für Nichtlineare Optik und Kurzzeitspektroskopie, 12489 Berlin, Germany

## Abstract

Quantum theory has linked microscopic currents and macroscopic polarizations of ferroelectrics, but the interplay of lattice excitations and charge dynamics on atomic length and time scales is an open problem. Upon phonon excitation in the prototypical ferroelectric ammonium sulfate [(NH_4_)_2_SO_4_], we determine transient charge density maps by femtosecond x-ray diffraction. A newly discovered low frequency-mode with a 3 ps period and sub-picometer amplitudes induces periodic charge relocations over some 100 pm, a hallmark of soft-mode behavior. The transient charge density allows for deriving the macroscopic polarization, showing a periodic reversal of polarity.

## I. INTRODUCTION

Ferroelectrics display a macroscopic electric polarization which originates from their peculiar electronic structure. Crystalline ferroelectric materials show a rich variety of lattice geometries and microscopic distributions of electronic charge within their unit cell.^1,2^ Extensive theoretical work has shown that, in contrast to early simplistic concepts, the macroscopic polarization **P**(**r**) cannot be derived solely from the time-independent microscopic electron density *ρ*(**r**).^3,4^ Instead, a *variation of charge density* as a function of an external parameter or a *current* is required. In theory, one has to calculate polarization *differences* between different states of the crystal from the respective electronic wavefunction. The elegant formalism presented in Refs. 4–7 expresses the polarization difference in terms of a quantum (Berry) phase calculated from the cell-periodic part of the electronic wavefunction.^8^ This method has been applied to calculate the stationary macroscopic polarization of a number of prototype ferroelectrics.

In contrast to such sophisticated treatments, experimental work benchmarking the theoretical predictions has remained scarce. Moreover, transient polarizations in ferroelectrics have been discussed in terms of nuclear motions in the crystal lattice, but the interplay of atomic displacements and electronic charge distributions is unknown.^9–11^ In this context, the pioneering concept of soft-modes which display a frequency strongly changing with charge relocations has played a key role.^12,13^ While soft-mode theory has qualitatively been invoked to explain the macroscopic properties of the ferro- and paraelectric phases of a material, the interplay of lattice motions and transient polarizations is not understood at the microscopic level.

Measurements of time-dependent microscopic charge densities have the potential to address such issues in a direct and quantitative way. X-ray powder diffraction with a femtosecond time resolution has been applied to determine transient charge density maps in crystalline materials.^14,15^ In a pump-probe approach, the excitation pulse provides a strong nonresonant electric field,^16^ induces lattice motions via resonant pumping,^17,18^ or a displacement of the nuclei upon electronic excitation.^19,20^ A synchronized hard x-ray probe pulse, diffracted from the excited sample, generates a Bragg diffraction pattern from which the momentary distribution of electronic charge density is derived. In a number of ionic materials, this method has provided detailed insight into charge relocations and their interplay with lattice motions.^16,20,21^

Here, ultrafast x-ray powder diffraction is applied for the first time to map the charge relocations connected with nonequilibrium low-frequency lattice excitations in a ferroelectric and to address the relation between transient microscopic electronic structure and macroscopic ferroelectric polarization. The prototype material ammonium sulfate [(NH_4_)_2_SO_4_, AS] is studied in the ferroelectric phase. The transient charge density maps in the electronically excited state of AS reveal periodic large-amplitude charge relocations upon small-amplitude soft-mode vibrations with a frequency of 12 cm^−1^ (0.36 THz) in the electronically excited state. The macroscopic electric polarization is derived from the transient microscopic charge density and exhibits a reversal of polarity with the 3-ps period of the soft-mode oscillation.

## II. EXPERIMENTAL METHODS

**FIG. 1.**
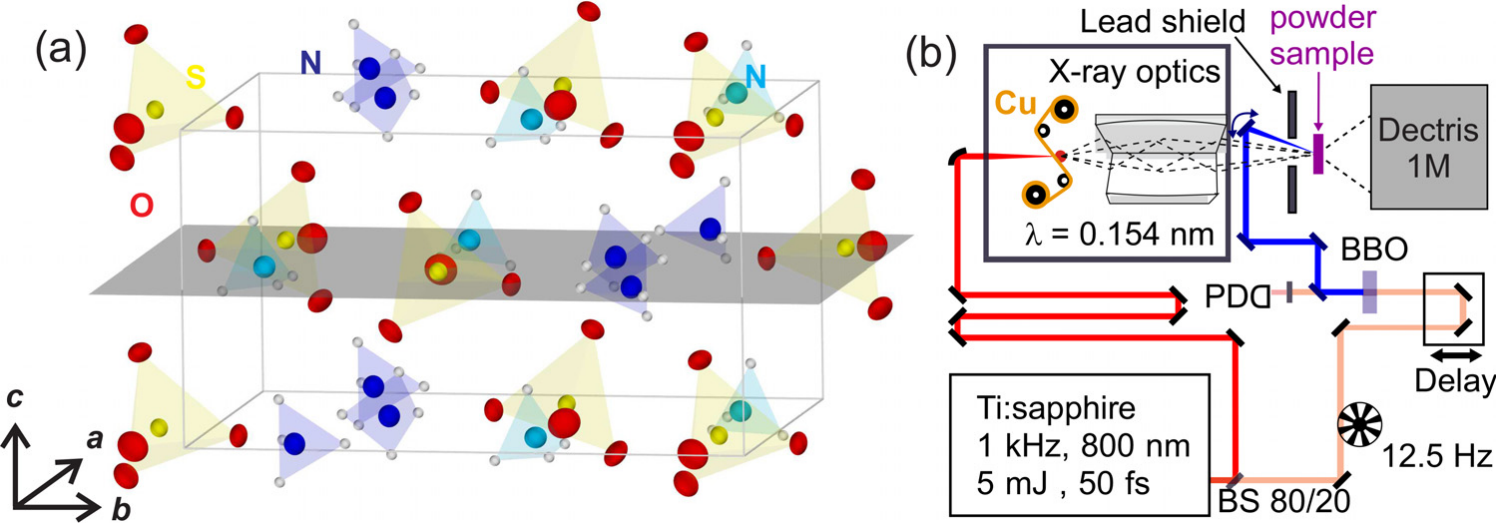
(a) Equilibrium crystal structure of ferroelectric AS with ${\text{NH}}_{4}^{+}$ tetrahedra (blue) and ${\text{SO}}_{4}^{2-}$ units (yellow). (b) Schematic drawing of the pump-probe set-up employed for the femtosecond diffraction experiment on powder samples. The samples are excited by 400 nm pulses generated by frequency doubling in a 250-*μ*m-thick BBO (type I phase-matching). The pump arm is mechanically chopped with 12.5 Hz and a photodiode (PD) determines at the 1 kHz repetition rate, i.e., for every x-ray probe pulse, if the sample was excited.

Ferroelectric AS crystallizes in an orthorhombic lattice structure (space group P*na*2_1_) with four formula units per unit cell [Fig. 1(a)].^22^ The dimensions of the ferroelectric unit cell are *a* = 0.78566(3) nm, *b *= 1.05813(4) nm, and *c* = 0.59530(2) nm. The experimentally observed ferroelectric polarization has a value of *P_fe_*(T = 222 K) = 6 mC m^−2^ slightly below the ferro- to paraelectric phase transition temperature T_*C*_ = 223 K.^23,24^ The AS samples were prepared fresh every day from finely ground commercially available starting material (Alfa Aesar, purity of 99.999%). Tightly pressed pellets with a thickness of ∼40 *μ*m were placed between a LiF entrance window (∼100 *μ*m thickness) and a polycrystalline diamond exit window (∼20 *μ*m thickness), which are acting as heat sinks, and fixed on a sample holder continuously rotating around an axis vertical to the x-ray beam. The sample temperature was set using a cooled nitrogen gas flow (Oxford Instruments CryojetHT). At the beginning of a measurement series, we slowly cooled down the sample under optical excitation and repeatedly recorded the diffraction pattern at different temperatures until the phase transition of the entire sample (at T_*C*_ = 223 K) was indicated by sudden changes of the angular position of selected Bragg reflections.^22^ The sample was then cooled down by another 20 K to achieve a uniform sample temperature of 200 ± 5 K under the experimental conditions. This is somewhat below the critical temperature T_*C*_ = 223 K of the ferro- to paraelectric phase transition of AS and, thus, the sample safely remains in the ferroelectric phase.^23–27^ An additional experiment was conducted at a sample temperature of ∼110 K, significantly lower than T_*C*_.

The ultrafast diffraction experiments are based on the optical pump/x-ray probe scheme illustrated in Fig. 1(b) where the sample is optically excited by 70 fs pulses with a center wavelength of 400 nm and probed with hard x-ray pulses similar to earlier experiments with paraelectric AS at room temperature.^20^ Both pump and x-ray probe pulses are derived from an amplified Ti:sapphire laser system delivering sub-50 fs pulses centered at 800 nm with an energy of 5 mJ and a repetition rate of 1 kHz. The optical pump pulses have an energy of 110 *μ*J and are focused to a spot size of ∼430 *μ*m, providing a peak intensity I_*p*_ ∼ 1 × 10^12^ W/cm^2^ at the sample surface. The sample is electronically excited via 3-photon absorption of the pump pulses. The major part (80%) of the 800 nm laser output is focused on a 20 *μ*m thin Cu tape target to generate hard x-ray pulses with a photon energy of 8.04 keV (Cu-K_*α*_) and a duration of roughly 100 fs.^16^ The emitted x-ray pulses are collected, monochromatized and focused to a ∼100 *μ*m spot size at the sample position by a Montel multilayer mirror (Incoatec) providing a flux ∼5 × 10^6^ photons/s. Further details of this table-top femtosecond hard x-ray source have been described earlier.^28^

**FIG. 2.**
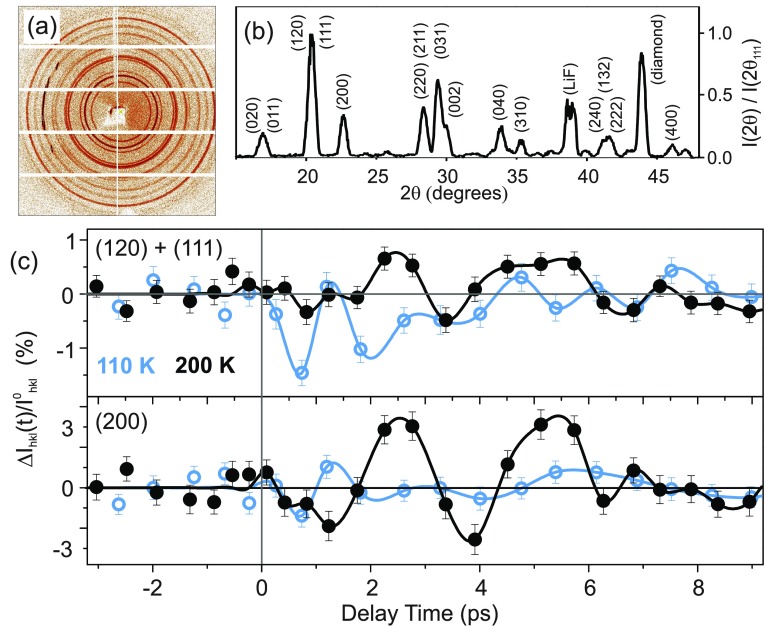
(a) Exemplary 2D powder diffraction pattern from unexcited ferroelectric AS at T = 200 K. (b) X-ray diffraction pattern obtained by integrating the 2D-pattern shown in (a) along the Debye-Scherrer rings. The normalized diffracted intensity is plotted as a function of the scattering angle 2*θ*, including assignments to lattice plains. (c) Change of diffracted x-ray intensity upon excitation $\Delta I_{hkl}(t)/I_{hkl}^{0}$ on two different Bragg reflections as a function of pump-probe delay in the femtosecond experiments at T = 200 K (black solid symbols) and T = 110 K (blue open symbols). The solid lines are guides to the eye.

The hard x-ray pulses serve for probing the pump-induced structural dynamics in the photoexcited sample. The Cu-K_*α*_ photons diffracted from the sample were recorded in transmission geometry by a large area detector (Pilatus Dectris 1M; pixel size 172 *μ*m × 172 *μ*m) which allows us to determine the intensity of multiple Debye-Scherrer rings at each delay time simultaneously. The pump pulses are chopped mechanically with a frequency of 12.5 Hz and a photodiode (PD) monitors for every x-ray probe pulse if the sample was excited. This experimental concept successfully mitigates the influence of temporal fluctuations of the femtosecond x-ray source on the signal-to-noise ratio, allowing for shot-noise limited measurements and the determination of the absolute change of diffracted intensity for each Bragg reflection.^29^ For each individual delay time, a total collection time of 140 s was selected, and ∼1000 different delays (i.e., ∼10 fs spacing in-between) within the chosen ∼12 ps long interval were measured in random order over 10 days. All-optical cross-correlations of the pump pulses with 800 nm pulses traveling along the optical path of the x-ray pulses were measured repeatedly to ensure a proper stacking of data from different days. Finally, we sorted all individual data points according to their delay time and then averaged ∼40 neighboring data points (i.e., a time interval of ∼400 fs) with increasing delay time together, to obtain transients with a good signal-to-noise ratio, as shown in Fig. 2(c).

## III. EXPERIMENTAL RESULTS

The powder diffraction pattern from an unexcited ferroelectric AS sample at T = 200 K is shown in Fig. 2(a). Integrating over all pixels with identical scattering angle 2*θ* yields 1D powder diffraction patterns as shown in Fig. 2(b) for an unexcited ferroelectric AS sample at T = 200 K. The result is in good agreement with literature and allows for an assignment of the 15 Bragg peaks to sets of lattice planes.^22^

Upon optical excitation, the angular positions of all observed reflections remain unchanged within the experimental accuracy. Furthermore, no additional Bragg reflections forbidden by the symmetry of the ferroelectric space group P*na*2_1_ are observed within our experimental sensitivity. Such observations confirm that the ferroelectric lattice geometry is preserved under our experimental conditions. In contrast, the diffracted intensities display pronounced changes Δ*I_hkl_*(*t*). In Fig. 2(c), the intensity change $\Delta I_{hkl}(t)/I_{hkl}^{0}=(I_{hkl}(t)-I_{hkl}^{0})/I_{hkl}^{0}$ on selected Bragg peaks is plotted as a function of pump-probe delay [$I_{hkl}(t)$, $I_{hkl}^{0}$: intensity diffracted with and without optical excitation]. The data recorded at T = 200 K exhibit pronounced oscillations of diffracted intensity with a period of ∼3 ps and a somewhat delayed onset. As also shown in Fig. 2(c), much faster and rapidly damped oscillations are observed at T = 110 K.

**FIG. 3.**
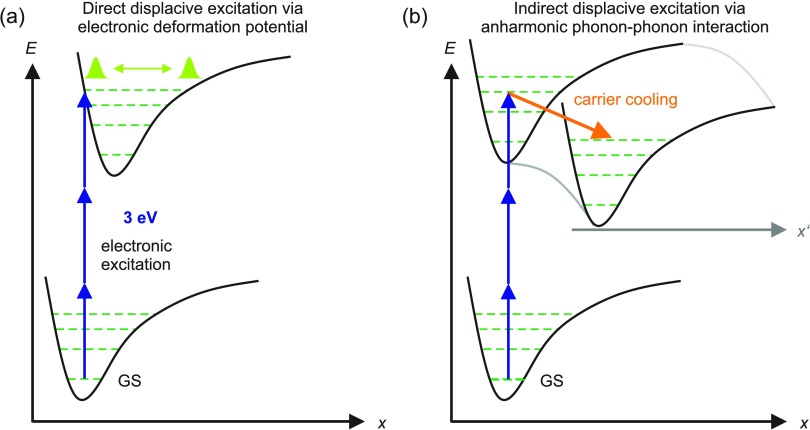
(a) Schematic drawing of the potential surfaces of the electronic ground and excited state along a low-frequency lattice coordinate. Optical excitation via 3-photon absorption can generate a displaced soft-mode wave packet in the electronically excited state of the crystal, thereby triggering a coherent soft-mode oscillation. (b) Even for zero displacement of the excited state potential, fast incoherent relaxation (orange arrow) on the multidimensional potential surface of the electronically excited state can generate a displaced soft-mode wave packet along the x′ coordinate after a time delay set by the relaxation kinetics.

The oscillations of diffracted intensity are due to coherent phonon excitations in the AS sample. There are different mechanisms for exciting coherent phonons as illustrated schematically in Fig. 3. Promotion to the electronically excited state results in a change of the electronic charge distribution and the generation of coherent nonequilibrium phonon displacements. A first mechanism is the direct displacive excitation of coherent optical phonons via the electronic deformation potential of the crystal. The electronic deformation potential in solids corresponds to an origin shift of the electronically excited potential surface vs. that of the electronic ground state as schematically depicted in Fig. 3(a). In this case, photoexcitation by a broadband femtosecond pulse generates a coherent superposition of vibrational quantum states in a quasi-instantaneous way. This nonstationary superposition is equivalent to wavepacket motions along the vibrational coordinate *x* and results in a cosine-like oscillation with respect to the minimum of the excited-state potential.^19,20^

In a different scenario illustrated in Fig. 3(b), low-frequency oscillations can also be induced impulsively by incoherent carrier relaxation on a time scale faster than the oscillation period. In this case, carrier relaxation excites a superposition of low-frequency quantum states either directly or via high-frequency excess phonons which couple anharmonically to low-frequency modes and, thus, induce their displacement. In contrast to the first mechanism, incoherent phonon excitation is possible even with a negligible shift of the excited state vs. ground state potential, and the subsequent electronic relaxation [orange arrow in Fig. 3(b)] leads to a temporally delayed onset of the phonon oscillation. Typical relaxation and, thus, onset times are in the range of up to a few picoseconds. The cosine-like oscillations in the experimental transients at T = 200 K do not set in immediately at *t* = 0 ps, as would be expected for a direct displacive excitation mechanism, but rather start with a 1–2 ps delay, which is clearly visible in Figs. 2(c) and 5. This suggests a predominance of the incoherent excitation mechanism under the present experimental conditions.

## IV. RECONSTRUCTION OF TRANSIENT ELECTRON DENSITY MAPS AND CHARGE DYNAMICS

The experimentally determined time-dependent intensity changes $\Delta I_{hkl}(t)/I_{hkl}^{0}$ are related to the transient x-ray structure factors *F_hkl_*(*t*) according to $\Delta I_{hkl}(t)/I_{hkl}^{0}=({\left|F_{hkl}(t)\right|}^{2}-{\left|F_{hkl}^{0}\right|}^{2})/{\left|F_{hkl}^{0}\right|}^{2}$, where $F_{hkl}^{0}$ are the known structure factors of the unperturbed material. The time-dependent electron density $\rho (r,t)=\eta \rho_{ex}(r,t)+(1-\eta )\rho_{0}(r)$ averaged over all crystallites and its change relative to the unperturbed electron density *ρ*_0_(**r**) of ferroelectric AS are extracted from the structure factors *F_hkl_*(*t*) by employing the maximum entropy method as implemented in the BayMEM suite of programs.^30–32^ The maximum entropy method maximizes the information entropy *S* which is defined as $S=-\sum_{\left(v=1\right)}^{N}\rho_{v}(t)\;\text{log}(\rho_{v}(t)/\rho_{0v})$, where the summation runs over a discretized grid of *N* voxels while fulfilling a set of constraints for the supplied structure factors *F_hkl_*(*t*).^30^ The fraction of excited unit cells *η* = 0.06 ± 0.03 is estimated from the absorbed pump fluence.^20^

**FIG. 4.**
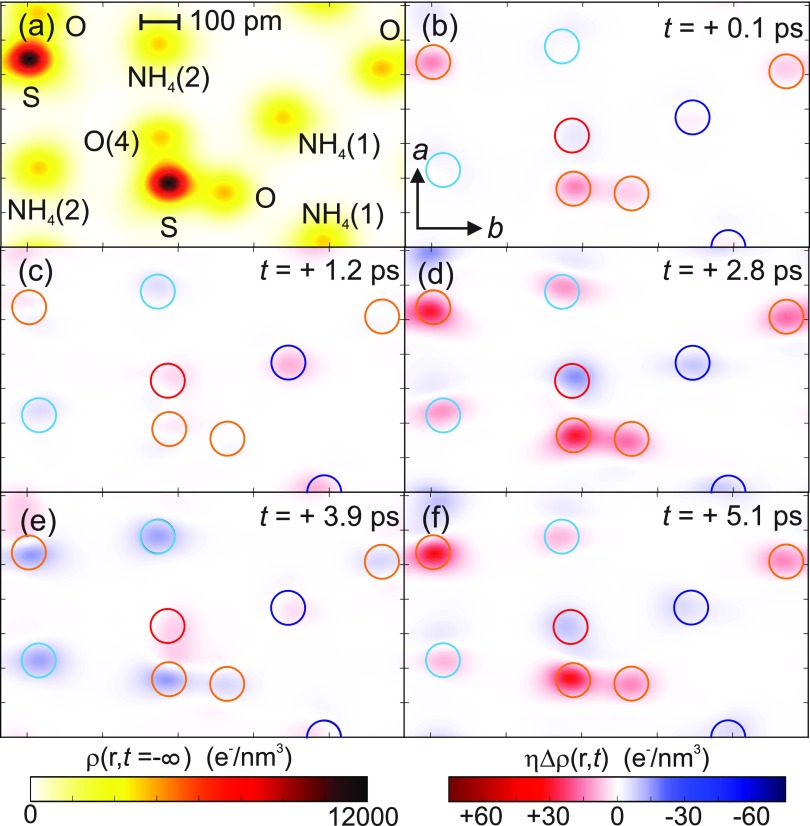
(a) Equilibrium electron density *ρ*_0_(**r**) as a two-dimensional contour map in the *z/c* = 0.5 plane. (b)–(f) Transient changes in electron density *η*Δ*ρ*(**r**, *t*) in the z/c = 0.5 plane [highlighted in Fig. 1(a)] at selected delay times. The positions of the atoms in the unexcited unit cell are indicated by colored circles.

In Fig. 4, equilibrium and transient charge density maps are summarized for the (*ab*)-lattice plane at *z/c *= 0.5 in the unit cell [highlighted in Fig. 1(a)]. Figure 4(a) shows the equilibrium charge density *ρ*_0_(**r**) and Figs. 4(b)–4(f) differential charge densities $\eta \Delta \rho (r,t)=\eta (\rho_{ex}(r,t)-\rho_{0}(r))=\rho (r,t)-\rho_{0}(r)$ for different pump-probe delays. The latter reveal a pronounced modulation of charge density with time, close to the original positions of the lattice atoms, which are indicated by colored circles. It is important to note that all major changes *η*Δ*ρ*(**r**, *t*) are centered on the ground state atomic positions and no charge transfer to previously unoccupied positions in space is observed. This behavior confirms the preservation of the ferroelectric lattice structure and is in striking contrast to paraelectric AS at T = 300 K where a spatial relocation of hydrogen atoms into a channel-like geometry along the *c-*axis has been observed.^20^ Within the ${\text{SO}}_{4}^{2-}$ anion, charge is shifted in a highly anisotropic way: the sulfur atom and three of the four oxygen atoms jointly exhibit a strong oscillatory increase in *η*Δ*ρ*(**r**, *t*), while the fourth oxygen atom O(4) behaves in an opposite fashion. Additionally, the net charge transfer from the ${\text{NH}}_{4}^{+}$ to the ${\text{SO}}_{4}^{2-}$ units enhances the local polarity in the crystal lattice compared with the stationary equilibrium structure.

**FIG. 5.**
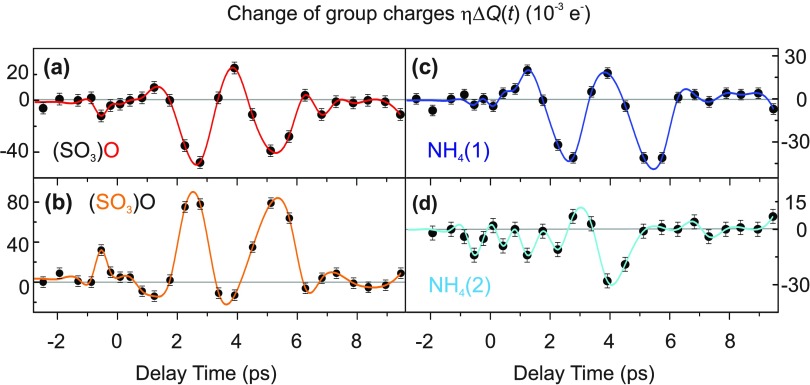
Spatially integrated group charges *η*Δ*Q*(*t*) of (a) the apical oxygen atom O(4) of the ${\text{SO}}_{4}^{2-}$ anion, (b) the SO_3_ unit, and (c) the first and (d) the second of the two crystallographically distinct ${\text{NH}}_{4}^{+}$ cations plotted as a function of pump-probe delay (black symbols). The colored lines are guides to the eye.

To derive changes of electronic charge *η*Δ*Q*(*t*) of different structural units, we subdivided the entire unit cell into subvolumes containing one atom each. This partitioning is based on the unperturbed crystal structure and each point in the unit cell is assigned to the atom nearest to it. Transient atomic charges were then obtained by integration over the respective subvolumes. In the case of the two ${\text{NH}}_{4}^{+}$ cations, the charge of the entire unit was added together. The results presented in Fig. 5 as a function of delay time reveal pronounced oscillatory changes of local charge with an oscillation period of ∼3 ps, corresponding to a frequency of 12 ± 2 cm^−1^ (0.36 ± 0.07 THz). The periodic charge modulations are due to coherent oscillations of a low-frequency lattice mode which is observed here for the first time and displays a frequency much lower than librational motions affecting the orientation of the ${\text{SO}}_{4}^{2-}$ and ${\text{NH}}_{4}^{+}$ units.^33–36^ These oscillations are severely damped on a time scale of several picoseconds. This is a known phenomenon in disordered powder samples, caused by inhomogeneous broadening of the frequencies of infrared-active phonons (in particular, the low-frequency soft-mode) compared with those measured in single crystals.^37,38^

The displacements of the 12 cm^−1^ mode are connected with a transfer of charge between the sulfur atom and three of the four oxygen atoms of the SO_4_ groups [Figs. 5(a) and 5(b)], and the apical oxygen atom O(4) of SO_4_ as well as one of the ${\text{NH}}_{4}^{+}$ cations [Figs. 5(c) and 5(d)]. The maximum amplitude of charge increase on the SO_3_ subunit [Fig. 5(b)] is close to the sum of the amplitudes of charge decrease on the O(4) atom and the NH_4_(1) unit. The second ${\text{NH}}_{4}^{+}$ cation displays much smaller changes of its associated charge [Fig. 5(d)]. These results give evidence of a net charge transfer to the SO_3_ unit over a distance of some 100 pm, i.e., a chemical bond length.

**FIG. 6.**
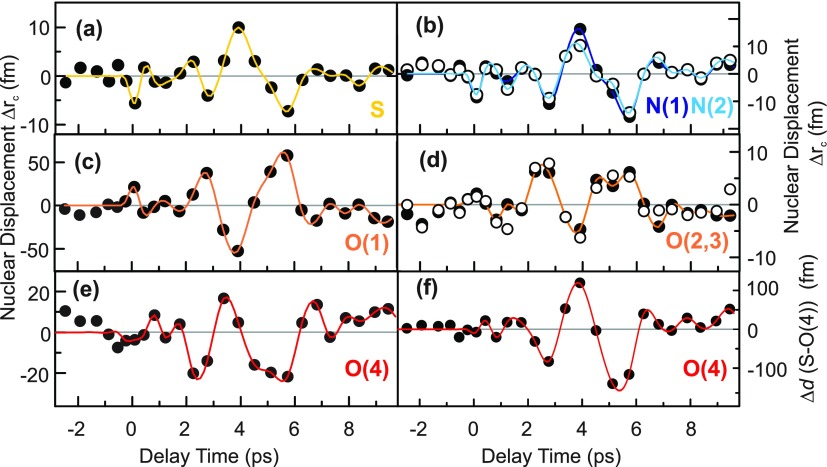
Transient nuclear displacements along the *c* axis Δ*r_c_*(*t*) of the (a) sulfur nucleus S, (b) two nitrogen nuclei N(1), N(2), and (c)–(e) four oxygen nuclei O(1), O(2), O(3), and O(4). (f) Transient change of the total bond length between the sulfur atom and the apical oxygen atom O(4). The colored lines are guides to the eye.

Transient atomic positions and interatomic distances are derived from an analysis of the core electron density of the sulfur, nitrogen, and oxygen atoms by fitting a three-dimensional Gaussian distribution to their high core electron density in the transient electron density maps. In contrast to valence electrons, the core electrons spatially follow the respective nucleus without any deformation of their electronic charge distribution. In conjunction with our limited experimental resolution, this gives an upper boundary for the magnitude of the nuclear motions. We find that the 12 cm^−1^ oscillations are connected with extremely small displacements of practically all heavy atoms in the unit cell, demonstrating a highly delocalized character of this lattice mode with shifts along the *a-, b-,* and *c-*axes. As an illustrative example, the bond length change Δ*d*(S-O_*ap*_) is plotted as a function of delay time in Fig. 6(f) and displays a magnitude of up to 100 fm, roughly 1000 times smaller than the S-O(4) distance. The nuclear displacements along the ferroelectric *c-*axis, shown in Figs. 6(a)–6(e), are in the sub-picometer regime as well.

The electrons of the hydrogen atoms also contribute to the total electron density map. However, due to the limited spatial resolution of the femtosecond x-ray diffraction experiment, there are no discernible electron density maxima at the proton positions and, thus, our experiment does not allow for an accurate determination of the transient proton positions within the ammonium ions. We, thus, assume in the following that the center of gravity of the 4 hydrogens in an ${\text{NH}}_{4}^{+}$ ion has a time-independent distance from the respective nitrogen nucleus and the protons follow the motion of the nitrogen nucleus.^39^

## V. DISCUSSION

The analysis of charge dynamics shows that electronic charge relocates during the coherent lattice motions over a distance 3 orders of magnitude larger than the individual nuclear displacements [cf. transient *η*Δ*ρ*(**r**) maps in Fig. 4]. This observation and the delocalized displacement pattern are a hallmark of soft-mode behavior, i.e., a very strong coupling of the vibrational and electronic degrees of freedom.^40^ This coupling results in strong local electric fields rearranging the electronic charge over large distances. Cochran's soft-mode theory,^12,13^ though not allowing for a quantitative description, predicts such a behavior to be particularly pronounced close to the critical temperature of a ferroelectric phase transition, in full agreement with our results. Thus, our results establish the soft-mode character of the newly discovered 12 cm^−1^ mode, occurring in the electronically excited state of AS.^40^

**FIG. 7.**
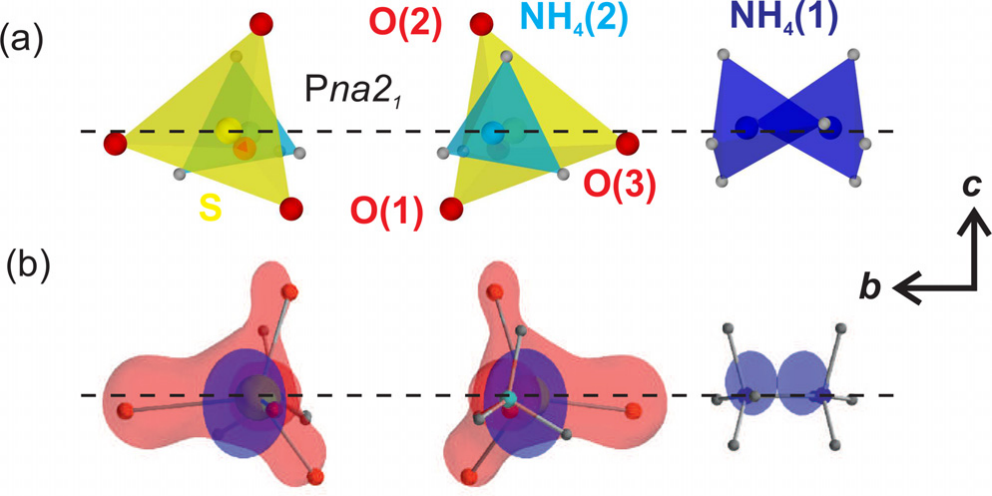
(a) Schematic drawing illustrating the orientation of the ${\text{SO}}_{4}^{2-}$ and ${\text{NH}}_{4}^{+}$ units along the *c*-axis in the ferroelectric phase of AS (space group P*na*2_1_). (b) Transient changes in the electron density *η*Δ*ρ*(**r**, *t*) in the *bc*-plane in an isosurface representation [*η*Δ*ρ*(**r**, *t*) = ±14 e/nm^3^ in red (increase) and blue (decrease)] at a delay time of 2.8 ps at T = 200 K viewed in the same orientation. The position of the atoms in the unexcited unit cell is indicated by colored spheres. The slightly darker red of the isosurface associated with the NH_4_(2) units is due to an overlap with other isosurfaces in this particular viewing direction.

A potential change of the macroscopic polarization Δ**P**(t) upon excitation of the microscopic soft-mode represents a key issue for the transient ferroelectric behavior. We recall that the electronically excited sample stays in the crystal structure of the ferroelectric phase. Correspondingly, the charge density projected onto the (*ab*) plane keeps the inversion symmetry during the soft-mode oscillations and therefore the polarization components in the (*ab*) plane vanish. On the other hand, an inversion center and an (*ab*) mirror plane perpendicular to the *c-*axis are absent. The tilt of the NH_4_ and SO_4_ tetrahedra relative to the *c-*axis, illustrated in Fig. 7(a), allows for a net electric dipole moment along *c*. Figure 7(b) displays transient changes *η*Δ*ρ*(**r**, *t*) of the electron density in the (*bc)* plane at T = 200 K for a pump-probe delay of 2.8 ps. The positions of all ${\text{NH}}_{4}^{+}$ and ${\text{SO}}_{4}^{2-}$ ions in the unit cell are projected on the *(bc)* plane (colored spheres).

In this representation, red/blue contours indicate the spatially resolved areas of charge increase/decrease. This illustrates again that the soft-mode relocates electronic charge within the ${\text{SO}}_{4}^{2-}$ units in a highly anisotropic fashion. The out-of-plane oxygen atom O(4) exhibits an oscillatory increase/decrease in charge density, while the sulfur atom and the three remaining oxygen atoms that lie roughly within the same *bc*-plane behave in an opposite fashion. Figure 7(b) also illustrates an additional and subtle, yet noticeable asymmetry along the polar c-axis that characterizes the redistribution of charge among the three oxygen atoms in the (*bc*) plane. This induces a time-dependent change of the macroscopic polarization Δ**P**(t) which is governed by the vibrational period of the 12 cm^−1^ mode.

It has been shown in the pioneering work of Resta and others that one cannot derive macroscopic electronic polarizations from the cell-periodic charge density maps *ρ*_0_(**r**) but only from changes of *ρ*(**r**, *λ*) as a function of an external parameter *λ*.^3–7^ In this context, the time-dependent charge density change *η*Δ*ρ*(**r**, *t*) averaged over all unit cells is particularly relevant because it provides the microscopic charge distribution as a function of the parameter time. The time-dependent charge density fulfills the continuity equation ∇·j(r,t)=−∂ρ(r,t)/∂t and allows for determining $\Delta P_{c}^{\text{electron}}(t)$ if one follows *ρ*(**r**, *t*) in small steps *dt* as a function of time. In practice, one solves the instantaneous Poisson equation $\Delta \phi (r,t)=-\rho (r,t)/\varepsilon_{0}$ from which the microscopic electric field E(r,t)=−∇ϕ(r,t) can be derived. After averaging the electric field over the unit cell $\tilde{E}(t)=V_{uc}^{-1}\int_{uc}dVE(r,t)$ the change of the macroscopic polarization is calculated from the difference $\Delta P_{c}^{\text{electron}}(t)=-\varepsilon_{0}[{\tilde{E}}_{z}(t)-{\tilde{E}}_{z}(t=-\infty )]$.

This general procedure can be further adopted in the special case of ferroelectric AS, since it consists of neutral molecular arrangements in layers parallel to the *ab*-plane of its unit cell, separated by regions of extremely low electron density. Microscopic currents flowing between one molecular layer and the neighboring layers can therefore be ruled out even during the soft-mode oscillation. In the language of Resta, this is called the Clausius-Mossotti-like case.^4^ In such a case, one can choose the (uneven) interface defined by the lowest electron density as a boundary between two adjacent subvolumes in *c* direction, over which the averaging of the electric field is then performed to calculate the change of the macroscopic polarization $\Delta P_{c}^{\text{electron}}(t)$.^41^

**FIG. 8.**
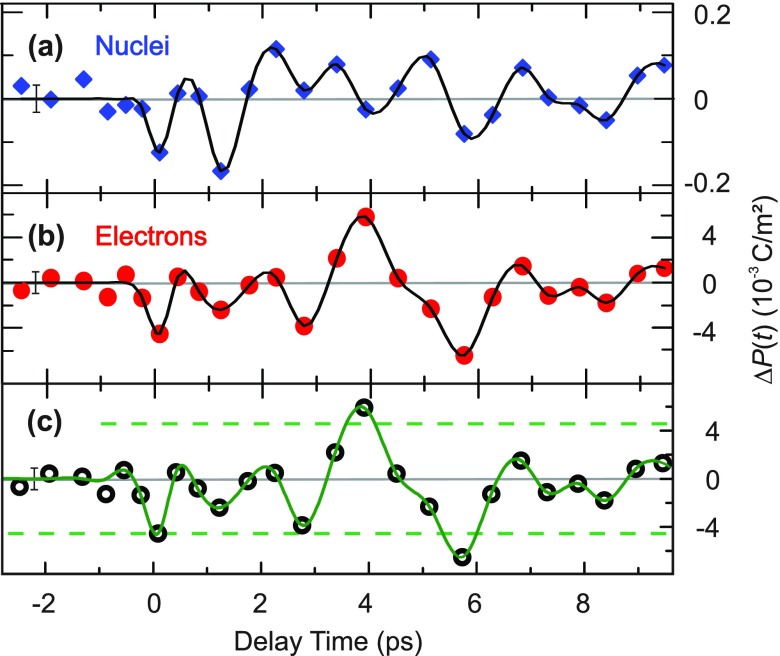
Time-dependent (a) nuclear and (b) electronic contributions to the (c) total change of the macroscopic electric polarization as derived from the transient charge density data (symbols, solid line). The dashed lines mark the amplitude of the equilibrium ferroelectric polarization at T = 200 K. The typical uncertainty for each case is indicated by an exemplary error bar.

The resulting total transient change of the macroscopic electric polarization parallel to the *c-*axis is given by $\Delta P(t)=\Delta P_{c}(t)=\Delta P_{c}^{\text{electron}}(t)+\Delta P_{c}^{\text{nuclei}}(t)$, where $\Delta P_{c}^{\text{electron}}(t)$ and $\Delta P_{c}^{\text{nuclei}}(t)$ represent the electronic and nuclear contributions to the polarization change. While the nuclear motions of the soft mode display a similar time evolution to the electronic polarization [Figs. 6(a)–6(e)], they are associated with only minute displacements of some tens of femtometers. Since these displacements are 3 orders of magnitude smaller than the typical distance of the electronic charge redistribution, the resulting total nuclear contribution to the overall polarization change [Fig. 8(a)] is roughly 30 times smaller than the electronic contribution [Fig. 8(b)], and can safely be neglected.

In Fig. 8(c), we show the resulting total transient change of the macroscopic electric polarization Δ*P_c_*(*t*) (symbols, solid line) as a function of time in comparison with the amplitude of the spontaneous ferroelectric polarization *P_fe_*(*T* = 200 K) ≃ 4.5 mC m^−2^ indicated by dashed lines.^24^ The amplitude of $\left|\Delta P_{c}(t)|\approx P_{fe}(T=200\;K\right)$ is close to the full ferroelectric polarization at a lattice temperature of 200 K. In other words, the tiny soft-mode displacements induce an oscillatory reversal of the full amplitude of electric polarization within an ultrashort 3 ps oscillation period. While the sign of *P_fe_* relative to the crystal structure has remained unknown,^24^ this phonon-driven reversal of electric polarization holds potential for switching applications with ultrahigh processing speed.

## VI. CONCLUSIONS

In conclusion, our results demonstrate the potential of ultrafast x-ray diffraction for unraveling the microscopic mechanisms behind ferroelectricity and for mapping the intrinsically ultrafast dynamics of electric polarizations upon phonon excitations. The fact that time-resolved charge density maps allow for solving the continuity equation establishes a natural and most direct link between microscopic charge density changes and macroscopic electric polarizations. This insight will allow for benchmarking *ab initio* quantum theory of ferroelectrics and will, thus, be instrumental for understanding and tailoring ferroelectric materials for a wide range of applications.
